# Comparative Efficacy of First‐Line Immune Checkpoint Inhibitor‐Based Combination Therapies in Patients With Sarcomatoid Renal Cell Carcinoma: A Japanese Multicenter Cohort Study

**DOI:** 10.1111/iju.70494

**Published:** 2026-05-09

**Authors:** Keita Tamura, Yuto Matsushita, Hiromitsu Watanabe, Takafumi Yanagisawa, Keiichiro Mori, Hirofumi Morinaka, Kensuke Bekku, Shingo Toyoda, Takuhisa Nukaya, Ryoichi Maenosono, Kazumasa Komura, Motoo Araki, Kazutoshi Fujita, Kiyoshi Takahara, Haruhito Azuma, Teruo Inamoto

**Affiliations:** ^1^ Department of Urology Hamamatsu University School of Medicine Shizuoka Japan; ^2^ Department of Urology Jikei University School of Medicine Tokyo Japan; ^3^ Department of Urology Kawasaki Medical School Okayama Japan; ^4^ Department of Urology Okayama University Graduate School of Medicine, Dentistry, and Pharmaceutical Sciences Okayama Japan; ^5^ Department of Urology Kindai University Faculty of Medicine Osaka Japan; ^6^ Department of Urology Fujita‐Health University School of Medicine Toyoake Aichi Japan; ^7^ Department of Urology Osaka Medical and Pharmaceutical University Osaka Japan

**Keywords:** dual immunotherapy, immune checkpoint inhibitor, immunotherapy plus tyrosine kinase inhibitor, sarcomatoid renal cell carcinoma

## Abstract

**Objectives:**

Sarcomatoid renal cell carcinoma (sRCC) is an aggressive histological variant associated with a poor prognosis. While immune checkpoint inhibitor (ICI)‐based combinations have become the standard of care, the optimal first‐line regimen, specifically dual immunotherapy (IO‐IO) vs. IO plus tyrosine kinase inhibitor (IO‐TKI), remains controversial. We herein examined the real‐world clinical outcomes of Japanese patients with sRCC.

**Methods:**

We conducted a retrospective multicenter study on 46 patients with advanced or metastatic sRCC receiving first‐line ICI‐based combination therapy between January 2018 and December 2024 (IO‐IO: *n* = 18; IO‐TKI: *n* = 28). In a comparative survival analysis, three favorable‐risk patients in the IO‐TKI group were excluded to align risk profiles, focusing on intermediate/poor‐risk groups (*n* = 43). The primary endpoint was overall survival (OS).

**Results:**

In the entire cohort (*n* = 46), the objective response rate was numerically higher in the IO‐TKI group (64.3%) than in the IO‐IO group (50.0%) (*p* = 0.37). In the comparative analysis of intermediate/poor‐risk patients (*n* = 43), progression‐free survival (PFS) was slightly longer (*p* = 0.071), and OS was significantly longer (*p* = 0.016) in the IO‐TKI group than in the IO‐IO group. A multivariable analysis adjusted for IMDC risk categories showed favorable survival with the IO‐TKI regimen (HR 0.37, *p* = 0.061).

**Conclusions:**

The present study indicates that first‐line IO‐TKI combination therapy represents a promising treatment option with a potential survival advantage over IO‐IO therapy for Japanese patients with sRCC. However, due to the retrospective design and small sample size, reliably determining the comparative efficacy of these regimens remains challenging, and further validation is warranted.

## 1 Introduction

Although sarcomatoid differentiation in renal cell carcinoma (RCC) is not a distinct histological subtype, it represents a high‐grade transformation that may occur in any RCC subtype, accounting for approximately 5%–8% of clear cell RCC (ccRCC), 8%–9% of chromophobe RCC, and 2%–3% of papillary RCC [1]. Despite its low incidence, sarcomatoid RCC (sRCC) is characterized by an aggressive clinical course, with a high propensity for early systemic metastasis and poor oncological outcomes [2]. Sarcomatoid features were previously shown to be present in approximately 20% of metastatic RCC (mRCC) cases, while up to 75% of RCC cases with sarcomatoid differentiation progressed to metastatic disease [3]. Histologically, sRCC is characterized by the presence of high‐grade spindle cell features resembling a primary sarcoma. These features are frequently associated with a distinct immune microenvironment, characterized by an increase in the expression of programmed death‐ligand 1 (PD‐L1) and the dense infiltration of tumor‐infiltrating lymphocytes [2]. These pathological hallmarks may contribute to the unique therapeutic sensitivities of sRCC, particularly to modern immunotherapies (IO), differentiating its clinical management from that of conventional RCC [4].

The therapeutic landscape for advanced or metastatic RCC has undergone a paradigm shift over the past decade, driven by the introduction of immune checkpoint inhibitors (ICI) and ICI‐based combination regimens that have reshaped the standard of care [5, 6]. Underpinning this shift, several landmark Phase 3 clinical trials have demonstrated that IO‐based systemic therapies, including dual IO (IO‐IO) or IO plus tyrosine kinase inhibitor (TKI) (IO‐TKI) therapy, provide superior clinical outcomes and overall survival (OS) to TKI monotherapy in patients with advanced or metastatic disease [6, 7]. Recent post hoc analyses of landmark phase III trials on patients with sRCC established the superiority of IO‐based systemic therapies over sunitinib. In the CheckMate 214 trial, nivolumab plus ipilimumab demonstrated a transformative survival benefit at the 5‐year follow‐up, achieving a median OS of 48.6 months vs. 14.2 months for sunitinib [a hazard ratio (HR) of 0.46 and objective response rate (ORR) of 60.8% vs. 23.0%] [8]. IO‐TKI combinations have shown similar outcomes: Lenvatinib plus pembrolizumab (the CLEAR trial) significantly improved progression‐free survival (PFS) (HR 0.39) and ORR (60.7% vs. 23.8%) [9], while nivolumab plus cabozantinib (CheckMate 9ER) [10] and avelumab plus axitinib (JAVELIN Renal 101) also yielded consistent clinical benefits [11]. Collectively, these findings justify the current paradigm shift, positioning IO‐based therapies as the standard of care for patients with sarcomatoid features.

However, few real‐world studies have examined therapeutic outcomes, and the relative efficacy of IO‐IO vs. IO‐TKI regimens in patients with sRCC remains unclear. In addition, existing evidence largely originates from Western populations, leaving a paucity of data on clinical outcomes among Asian patients with sRCC. Therefore, the objective of the present study was to examine the clinical outcomes of Japanese patients with advanced or metastatic sRCC treated with different first‐line IO‐based systemic therapies in a real‐world setting.

## 2 Patients and Methods

### 2.1 Study Design and Patients

This study was approved by the Institutional Review Board of the principal institution (Hamamatsu University School of Medicine, Hamamatsu, Japan; approval number: 25–034). We conducted a retrospective multicenter study on 656 patients with advanced or mRCC who received ICI–based combination therapies at eight academic institutions and their affiliated hospitals in Japan between January 2018 and December 2024. Patients selected for this study had histologically verified sRCC and were treated with first‐line IO‐IO or IO‐TKI therapy. We ultimately included 46 patients diagnosed with advanced or metastatic sRCC in this study: 18 treated with IO‐IO and 28 with IO‐TKI. The need to obtain informed consent from all patients included in this study was waived due to its retrospective study design.

### 2.2 Treatment With ICI–Based Combination Agents

In this series, 1 of the 5 ICI–based regimens (IO‐IO: Nivolumab plus ipilimumab; IO‐TKI: Pembrolizumab plus axitinib, avelumab plus axitinib, nivolumab plus cabozantinib, or pembrolizumab plus lenvatinib) currently available in Japanese clinical practice was administered to all patients as first‐line therapy against sRCC under standard dosing schedules, as previously reported [8, 9, 10, 11, 12]. However, according to the product label, dose modifications to each agent were permitted based on the severity of adverse events.

### 2.3 Study Endpoints

The present study had two main objectives. The first aim was to evaluate real‐world clinical outcomes, including OS, PFS, and ORR, in patients with sRCC treated with first‐line ICI‐based regimens. The second aim and primary comparative goal was to assess the relative efficacy of IO‐IO vs. IO‐TKI combinations. To perform this specific comparison, we focused on intermediate‐ and poor‐risk groups to align with therapeutic indications and minimize selection bias. Secondary endpoints included the identification of clinical and pathological factors that affect prognosis. Through these analyses, we aimed to clarify the optimal therapeutic strategy for this highly aggressive RCC variant.

### 2.4 Evaluation

Clinicopathological data were collected from medical records. Prior to the initiation of first‐line ICI‐based therapy, the Performance Status (PS) was assessed by the Karnofsky PS (KPS) scale, and laboratory data were measured using standard methods. Baseline radiological assessments included computed tomography (brain, chest, abdomen) and/or bone scanning. Based on the outcomes of these evaluations, a risk classification for each patient was conducted using International Metastatic RCC Database Consortium (IMDC) models [13].

As a rule, tumor measurements were performed with computed tomography every 12 weeks following the introduction of first‐line ICI‐based therapy. OS was defined as the interval between the start of first‐line therapy and the date of death from any cause or censoring on the day of the last follow‐up visit. PFS was defined as the time from the initiation of first‐line therapy to the date of radiological progression or death from any cause.

### 2.5 Statistical Analysis

Survival outcomes were calculated using the Kaplan–Meier method and compared with the Log‐rank test. The prognostic significance of specific factors was analyzed employing uni‐ and multivariable Cox proportional hazards models, with results being presented as HRs and corresponding 95% confidence intervals (CIs). In comparisons of baseline patient characteristics between treatment groups, Fisher's exact test was used for categorical variables, while the Mann–Whitney *U* test was employed for continuous variables. All statistical analyses were performed using EZR software (Saitama Medical Center, Jichi Medical University, ver. 1.70) [14], and a *p*‐value < 0.05 was considered to be significant.

## 3 Results

### 3.1 Baseline Patient Characteristics of the Overall Cohort

**FIGURE 1:**
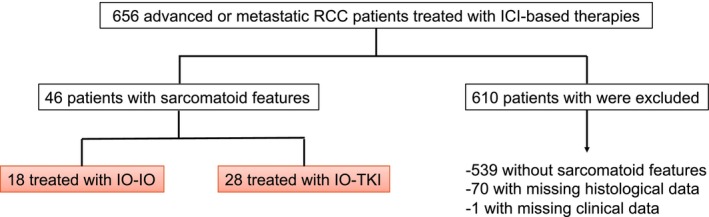
Flowchart of patient selection. Of 656 patients with metastatic renal cell carcinoma (mRCC) treated with ICI‐based combination regimens, 539 patients with non‐sarcomatoid histology, 70 patients with insufficient histological documentation or pathological description, and 1 patient with a missing clinical description were excluded. The final analysis included 46 patients with histologically confirmed sarcomatoid features, categorized into those receiving IO–IO (*n* = 18) and IO–TKI (*n* = 28) combination therapies. ICI, immune checkpoint inhibitor; IO, immunotherapy; TKI, tyrosine kinase inhibitor.

The patient selection process is summarized in Figure 1. Forty‐six patients with advanced or metastatic RCC and histologically confirmed sarcomatoid differentiation were included in the final analysis. The baseline characteristics of the overall cohort are shown in Table 1. Briefly, 76.1% of patients were male, and 10.9% had a KPS of < 80. Diagnostic specimens confirming sarcomatoid features were obtained via biopsy in 30.4% of cases and nephrectomy in 69.6%. The clear cell carcinoma subtype was predominant (65.2%).

**TABLE 1:** Patient characteristics of the overall cohort.

	*N* = 46
Sex, *n* (%)	
Male	35 (76.1)
Female	11 (23.9)
Age at start of first‐line therapy, years Median (IQR)	65 (55–72)
BMI ≥ 25 kg/m^2^, *n* (%)	18 (39.1)
KPS < 80%, *n* (%)	5 (10.9)
Diagnostic specimen, *n* (%)	
Biopsy	14 (30.4)
Partial nephrectomy	4 (8.7)
Radical nephrectomy	28 (60.9)
Previous nephrectomy, *n* (%)	32 (69.6)
Histological subtype, Clear cell carcinoma, *n* (%)	30 (65.2)
Synchronous metastasis, *n* (%)	27 (58.7)
Multi‐organ metastases, *n* (%)	21 (45.7)
Lymph node metastasis, *n* (%)	17 (37.0)
Metastatic sites, *n* (%)	
Lung	28 (60.9)
Bone	8 (17.4)
Liver	3 (6.5)
Brain	2 (4.4)
IMDC risk group, *n* (%)	
Favorable‐risk	3 (6.5)
Intermediate‐risk	27 (58.7)
Poor‐risk	16 (34.8)
Type of first‐line targeted therapy, *n* (%)	
Ipilimumab + Nivolumab	18 (39.1)
Pembrolizumab + Axitinib	9 (19.6)
Avelumab + Axitinib	5 (10.9)
Nivolumab + Cabozantinib	7 (15.2)
Pembrolizumab + Lenvatinib	7 (15.2)

Abbreviations: BMI, body mass index; ICI, immune checkpoint inhibitor; IMDC, International Metastatic Renal Cell Carcinoma Database Consortium; IO, immunotherapy; IQR, interquartile range; KPS, Karnofsky performance status; TKI, tyrosine kinase inhibitor.

Regarding disease extent, 58.7% of patients presented with synchronous metastasis, and 45.7% had multi‐organ metastases. Based on the IMDC risk classification, patients were categorized as favorable (6.5%), intermediate (58.7%), and poor‐risk (34.8%). First‐line systemic therapies consisted of IO‐IO (*n* = 18 [39.1%]) and IO‐TKI combinations (*n* = 28 [60.9%]). The median follow‐up duration for the entire cohort was 20.3 months (interquartile range, 8.2–33.7).

### 3.2 Efficacy and Survival Outcomes in the Overall Cohort

**FIGURE 2:**
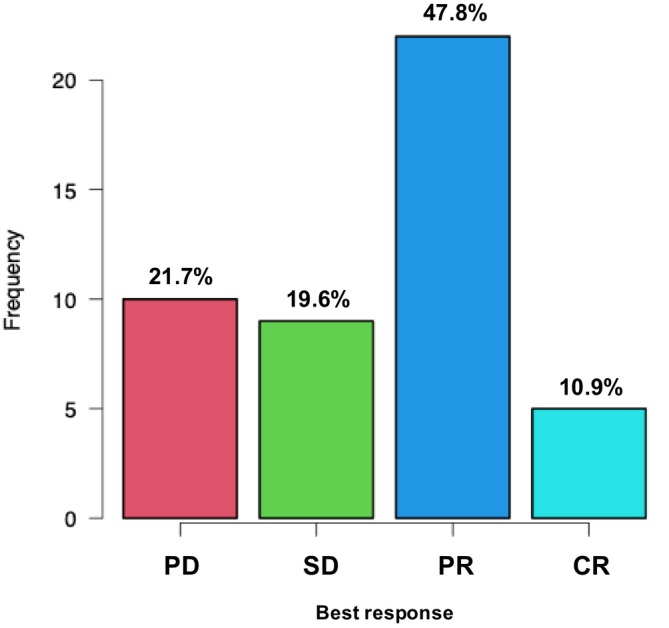
Best clinical response rate for the entire cohort. The bar chart represents the frequency and percentage of patients in each response category (*n* = 46). CR, complete response; PD, progressive disease; PR, partial response; SD, stable disease.

The best overall response rate for the entire cohort (*n* = 46) is summarized in Figure 2. Among evaluable patients, the ORR was 58.7% (27/46), with 10.9% (5/46) achieving a complete response (CR) and 47.8% (22/46) achieving a partial response (PR). Stable disease and progressive disease were observed in 19.6% (9 patients) and 21.7% (10 patients), respectively, resulting in a disease control rate of 78.3%.

**FIGURE 3:**
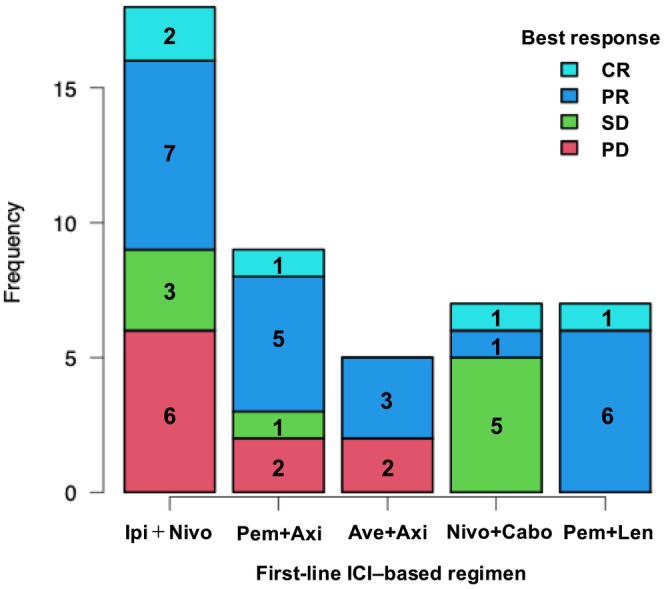
Summary of best overall responses for each ICI–based combination regimen. A horizontal stacked bar chart illustrating the distribution of best overall responses across all evaluated treatment strategies (IO–IO and four types of IO–TKI combinations). The numbers within the bars indicate the absolute number of patients in each response category. Ave, avelumab; Axi, axitinib; Cabo, cabozantinib; CR, complete response; ICI, immune checkpoint inhibitor; IO, immunotherapy; Ipi, ipilimumab; Len, lenvatinib; Nivo, nivolumab; PD, progressive disease; Pem, pembrolizumab; PR, partial response; SD, stable disease; TKI, tyrosine kinase inhibitor.

The clinical outcomes of each regimen are visualized using a stacked bar chart in Figure 3. The ORR was 50.0% (2 CRs and 7 PRs) in the IO–IO group (*n* = 18) and 64.3% (3 CRs and 15 PRs) in the IO–TKI group (*n* = 28). While the IO–TKI group showed a numerically higher ORR, no significant difference was observed between the two treatment groups (*p* = 0.37). The pembrolizumab plus lenvatinib subgroup showed a highly concentrated density at the PR level, achieving an ORR of 100% (1 CR and 6 PRs).

**FIGURE 4:**
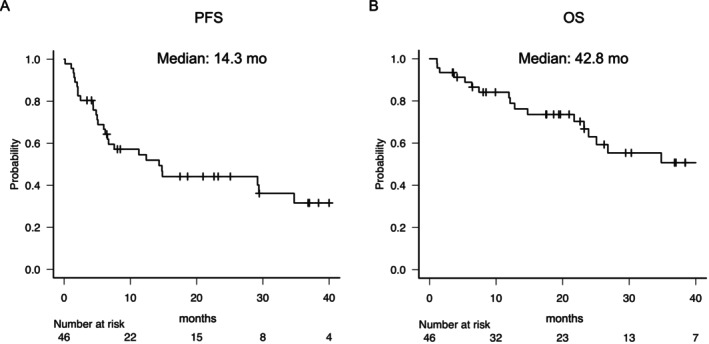
Kaplan–Meier curves for survival outcomes in the entire cohort. (A) Progression‐free survival and (B) overall survival for all evaluable patients with sRCC receiving first‐line ICI‐based therapy (*n* = 46). ICI, immune checkpoint inhibitor; OS, overall survival; PFS, progression‐free survival; sRCC, sarcomatoid renal cell carcinoma.

Survival outcomes for the entire cohort (*n* = 46) are shown in Figure 4. Median PFS was 14.3 months (CI, 6.21–34.7), with estimated 2‐ and 3‐year PFS rates of 44.2% and 31.6%, respectively. Regarding OS, median OS was 42.8 months (95% CI, 23.2–NR). Estimated 2‐ and 3‐year OS rates were 63.0% and 50.8%, respectively.

### 3.3 Comparison of IO‐IO and IO‐TKI Groups in the Intermediate/Poor‐Risk Cohort

To precisely evaluate the comparative efficacy of the IO‐IO and IO‐TKI regimens, we focused on a comparative cohort of 43 patients, excluding favorable‐risk patients. The baseline characteristics of this comparative cohort (IO‐IO, *n* = 18; IO‐TKI, *n* = 25) are shown in Table 2. While the proportion of poor‐risk patients tended to be higher in the IO‐IO group than in the IO‐TKI group (*p* = 0.056), there were no statistically significant imbalances between the two groups regarding other key prognostic factors, including KPS < 80 (*p* = 1.000) and the presence of multi‐organ metastases (*p* = 1.000). Furthermore, there was no significant difference in the median follow‐up duration between the IO‐IO group (17.3 months) and the IO‐TKI group (19.7 months) (*p* = 0.32).

**TABLE 2:** Patient characteristics of the comparative cohort.

	IO‐IO (*n* = 18)	IO‐TKI (*n* = 25)	*p*
Sex, *n* (%)			
Male	14 (77.8)	18 (72.0)	0.74
Female	4 (22.2)	7 (28.0)	
Age at start of first‐line therapy, years Median (IQR)	64 (54.5–71.8)	66 (55.0–72.0)	0.99
BMI ≥ 25 kg/m^2^, *n* (%)	5 (27.8)	11 (44.0)	0.35
KPS < 80%, *n* (%)	2 (11.1)	3 (12.0)	1
Previous nephrectomy, *n* (%)	10 (55.6)	19 (76.0)	0.20
Histological subtype, Clear cell carcinoma, *n* (%)	9 (50.0)	18 (72.0)	0.20
Synchronous metastasis, *n* (%)	11 (61.1)	16 (64.0)	1
Multi‐organ metastases, *n* (%)	9 (50.0)	12 (48.0)	1
Lymph node metastasis, *n* (%)	9 (50.0)	7 (28.0)	0.20
Metastatic sites, *n* (%)			
Lung	10 (55.6)	17 (68.0)	0.52
Bone	3 (16.7)	4 (16.0)	1
Liver	2 (11.1)	1 (4.0)	0.56
Brain	2 (11.1)	0 (0)	0.17
IMDC risk group, *n* (%)			
Intermediate‐risk	8 (44.4)	19 (76.0)	0.056
Poor‐risk	10 (55.6)	6 (24.0)	
Type of first‐line targeted therapy, *n* (%)			
Ipilimumab + Nivolumab	18 (100)	0 (0)	< 0.001
Pembrolizumab + Axitinib	0 (0)	7 (28.0)	
Avelumab + Axitinib	0 (0)	5 (20.0)	
Nivolumab + Cabozantinib	0 (0)	6 (24.0)	
Pembrolizumab + Lenvatinib	0 (0)	7 (28.0)	
Follow‐up period, months Median (IQR)	17.3 (6.4–23.7)	19.7 (11.9–36.8)	0.32

Abbreviations: BMI, body mass index; ICI, immune checkpoint inhibitor; IMDC, International Metastatic Renal Cell Carcinoma Database Consortium; IO, immunotherapy; IQR, interquartile range; KPS, Karnofsky performance status; TKI, tyrosine kinase inhibitor.

**FIGURE 5:**
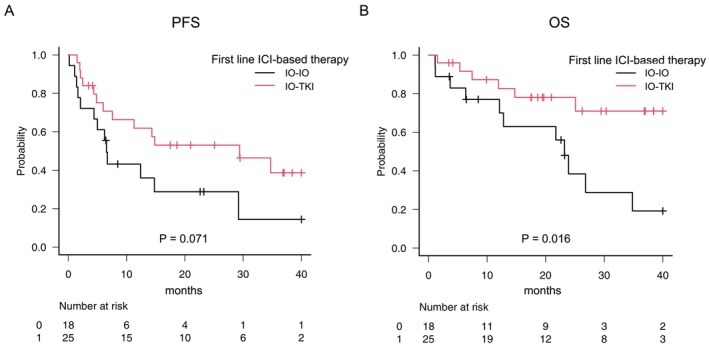
Kaplan–Meier curves for survival outcomes in patients with intermediate or poor IMDC risk. (A) Progression‐free survival and (B) overall survival compared between the IO–IO (*n* = 18) and IO–TKI (*n* = 25) groups. *p*‐values were calculated using the log‐rank test. The number of patients at risk is indicated below each plot. ICI, immune checkpoint inhibitor; IMDC, International Metastatic Renal Cell Carcinoma Database Consortium; IO, immunotherapy; OS, overall survival; PFS, progression‐free survival; TKI, tyrosine kinase inhibitor.

Within this comparative cohort, we compared survival outcomes between the IO‐IO and IO‐TKI groups. PFS was longer in the IO–TKI group than in the IO–IO group (*p* = 0.071) (Figure 5A). OS was significantly longer in the IO–TKI group than in the IO–IO group (*p* = 0.016) (Figure 5B). While median OS was not reached in the IO–TKI group, it was 23.2 months in the IO–IO group throughout the follow‐up period. To confirm these results, we performed an exploratory subgroup analysis stratified by the IMDC risk category (intermediate vs. poor), as shown in Figure S1. Despite the limited sample size, survival in both the intermediate‐ and poor‐risk categories was numerically longer in the IO‐TKI group than in the IO‐IO group.

Regarding subsequent treatments in this cohort, 17 patients (94.4%) in the IO‐IO group and 18 patients (72.0%) in the IO‐TKI group discontinued first‐line therapy, primarily due to disease progression or adverse events. Subsequently, 7 patients (38.9%) in the IO‐IO group and 10 patients (40.0%) in the IO‐TKI group received second‐line systemic therapies. The most common subsequent agent was cabozantinib (3 patients in the IO‐IO group and 8 in the IO‐TKI group), followed by pazopanib (3 in the IO‐IO group and 1 in the IO‐TKI group).

### 3.4 Prognostic Factors for Overall Survival

To identify predictors of OS following the initiation of first‐line ICI‐based therapy in 43 sRCC patients, uni‐ and multivariable Cox proportional hazards analyses of several potential parameters were performed (Table 3). The univariable analysis revealed that OS correlated with sex, age, KPS < 80, lymph node metastasis, and type of first‐line therapy. To identify independent prognostic factors for OS while adjusting for baseline imbalances, we performed a multivariable analysis (Table 3). Due to the limited number of events (*n* = 17), we employed a parsimonious model to prevent statistical overfitting, incorporating two clinically essential variables: The IMDC risk category (poor vs. intermediate) and treatment regimen (IO–TKI vs. IO–IO). After adjusting for the IMDC risk category, the risk of death continued to be lower in the IO–TKI group than in the IO–IO group, with an adjusted HR of 0.37 (95% CI, 0.13–1.05; *p* = 0.061). In contrast, the IMDC risk category was not identified as a significant independent prognostic factor in this multivariable model (HR 1.66; 95% CI, 0.61–4.54; *p* = 0.32).

**TABLE 3:** Univariable and multivariable Cox proportional hazards analyses of factors associated with overall survival after the introduction of first‐line ICI‐based therapy for sRCC patients in the intermediate or poor IMDC category.

	Univariable analysis		Multivariable analysis	
	HR (95% CI)	*p*	HR (95% CI)	*p*
Sex (Female vs. male)	2.99 (1.12–7.97)	0.028		
Age (continuous)	0.95 (0.90–0.99)	0.025		
BMI, kg/m^2^ (≥ 25 vs. < 25)	0.83 (0.29–2.39)	0.73		
KPS, % (< 80 vs. ≥ 80)	3.75 (1.00–14.1)	0.049		
Previous nephrectomy (yes vs. no)	0.83 (0.31–2.18)	0.70		
Histological subtype (non‐Clear cell vs. Clear cell)	0.95 (0.35–2.58)	0.93		
Metastasis (synchronous vs. metachronous)	2.72 (0.78–9.48)	0.18		
Multi‐organ metastases (yes vs. no)	1.93 (0.71–5.23)	0.20		
Lymph node metastasis (yes vs. no)	3.05 (1.15–8.07)	0.025		
Lung metastasis (yes vs. no)	0.89 (0.34–2.34)	0.81		
Bone metastasis (yes vs. no)	1.24 (0.40–3.84)	0.72		
Liver metastasis (yes vs. no)	4.48 (0.96–20.0)	0.057		
Brain metastasis (yes vs. no)	1.26 (0.16–9.75)	0.82		
IMDC risk group (poor vs. intermediate)	2.28 (0.88–5.95)	0.090	1.66 (0.61–4.54)	0.32
First‐line therapy (IO‐TKI vs. IO‐IO)	0.31 (0.11–0.85)	0.022	0.37 (0.13–1.05)	0.061

Abbreviations: BMI, body mass index; CI, confidence interval; HR, hazard ratio; ICI, immune checkpoint inhibitor; IMDC, International Metastatic Renal Cell Carcinoma Database Consortium; IO, immunotherapy; KPS, Karnofsky performance status; sRCC, sarcomatoid renal cell carcinoma; TKI, tyrosine kinase inhibitor.

## 4 Discussion

During the last decade, the introduction of ICIs has markedly changed the therapeutic landscape for patients with mRCC [5, 6]. However, due to the aggressive nature and rarity of sarcomatoid differentiation, therapeutic strategies for sRCC have become more complex, and the optimal first‐line regimen, specifically, the choice between IO‐IO and IO‐TKI, remains controversial [15]. Accordingly, it is extremely important to evaluate real‐world outcomes to guide the selection of optimal agents for this specific cohort. In the present study, we retrospectively assessed the clinical outcomes of 46 sRCC patients treated with first‐line ICI‐based combination therapies.

In this series, the IO‐TKI group demonstrated favorable oncological outcomes, achieving a significantly superior OS and numerically higher ORR of 64.3%, in contrast to 50.0% in the IO‐IO group. While the difference in PFS was marginal (*p* = 0.071), the significant improvement in OS (*p* = 0.016) indicates a potential therapeutic benefit of the IO‐TKI combination for patients with sRCC. The univariate analysis identified the treatment regimen (IO‐TKI) as a significant predictor of longer OS (*p* = 0.022), and this advantage was still observed in the multivariable analysis adjusted for the IMDC risk category (HR 0.37, *p* = 0.061). However, it is necessary to discuss the potential reasons behind the survival advantage observed in the IO‐TKI group. A critical factor to consider is the potential imbalance in baseline characteristics. For instance, although brain metastasis is generally associated with poor prognosis in metastatic RCC, its incidence was similar between the groups in our cohort (IO‐IO, 11.1% vs. IO‐TKI, 12.0%; *p* = 0.17). This suggests that its confounding effect on the observed overall survival outcomes was likely minimal. Conversely, even after the exclusion of favorable‐risk patients, the percentage of IMDC poor‐risk patients tended to be higher in the IO‐IO group (55.6%) than in the IO‐TKI group (24.0%) (*p* = 0.056). This result reflects real‐world clinical practice, where physicians generally select nivolumab plus ipilimumab for patients with aggressive disease or a poor PS based on evidence from the CheckMate 214 trial [8]. Therefore, superior OS in the IO‐TKI group may be partially attributed to the higher percentage of poor‐risk patients in the IO‐IO group. However, the IO‐TKI regimen maintained a favorable HR even after statistical adjustments for IMDC risk categories. This result suggests that the distinct therapeutic benefit provided by IO‐TKI plays a significant role in controlling sRCC.

A number of studies have investigated the efficacy of systemic therapies for sRCC. Post hoc analyses of landmark phase III trials, such as CheckMate 214, CLEAR, and JAVELIN Renal 101, have consistently demonstrated the clinical benefits of ICI‐based combinations over sunitinib [8, 9, 11]. Furthermore, a recent network meta‐analysis comprising 568 sRCC patients confirmed the efficacy of various first‐line ICI‐based combinations. Importantly, the present study highlighted distinct therapeutic profiles; while nivolumab plus ipilimumab was associated with the highest likelihood of achieving CR, the IO‐TKI combination (specifically nivolumab plus cabozantinib) demonstrated the highest probability of prolonging both PFS and OS [16]. In our real‐world Japanese cohort, CR rates differed numerically between treatment regimens; however, the small sample size precluded definitive conclusions.

It is of interest to compare our real‐world results with these landmark studies. The ORR of 64.3% in the IO‐TKI group is similar or even superior to the rates observed in these phase III trials (range 46.8%–60.7%) [8, 9, 10, 11, 12]. Furthermore, in terms of real‐world evidence, the ARON‐1 study, a large international multicenter analysis, reported median OS of 34.4 months for IO‐TKI and 26.4 months for IO‐IO, with no significant difference between the two regimens (*p* = 0.729) [17]. Similarly, a recent Canadian multicenter study demonstrated that among patients with sarcomatoid features, the IO‐TKI group exhibited numerically longer median OS than the IO‐IO group (48 vs. 36 months) [3]. Collectively, these findings suggest that while optimal sequencing continues to be debated, IO‐TKI regimens provide rapid and robust disease control, which is beneficial for aggressive variants such as sRCC.

Another point of interest focuses on the biological rationale for selecting treatment strategies for this specific population. Sarcomatoid tumors are characterized by an inflammatory tumor microenvironment with high PD‐L1 expression (approx. 50%) and dense tumor‐infiltrating lymphocytes [8, 18]. Theoretically, these pathological features favor dual checkpoint blockade (IO‐IO), such as nivolumab plus ipilimumab. In contrast, sRCC exhibits lower angiogenic gene expression and resistance to vascular endothelial growth factor receptor‐targeted therapy, thereby limiting the therapeutic efficacy of TKI monotherapy [19].

In general RCC models, it has been proposed that VEGF inhibitors can reprogram the immunosuppressive microenvironment by reducing the accumulation of myeloid‐derived suppressor cells and regulatory T cells, providing a strong rationale for IO‐TKI combinations [20, 21]. Accordingly, the dual inhibition of immune checkpoints and angiogenesis in IO‐TKI combinations may offer a potent synergistic effect, which supports the favorable survival outcome [22].

However, recent transcriptomic analyses, such as those by Bakouny et al., have robustly demonstrated that in the specific context of sRCC, immune‐related gene signatures, rather than angiogenesis‐related genes, are predominantly expressed [23]. This biological reality questions the uniform efficacy of classical anti‐angiogenic therapies in this variant. In fact, while IO‐TKI combinations showed promising overall clinical benefits in our cohort, we observed notable heterogeneity in therapeutic responses depending on the specific combination regimen. As our data demonstrated, PD was observed in patients treated with axitinib‐based combinations, whereas no PD cases were observed in the cabozantinib‐ or lenvatinib‐based groups. This clinical heterogeneity aligns with the transcriptomic findings of sRCC, suggesting that the underlying mechanisms are more complex than simple VEGF inhibitor‐mediated normalization of the tumor vasculature. It is plausible that multi‐targeted TKIs, such as cabozantinib (which also targets MET and AXL) or lenvatinib (which targets FGFR), exert distinct immunomodulatory effects on the sarcomatoid microenvironment compared to more selective VEGFR inhibitors like axitinib. Further translational studies are needed to fully elucidate the optimal therapeutic targets and the exact mechanisms of specific TKI agents in this highly immune‐infiltrated variant.

The present study has several limitations. First, the retrospective design and small sample size (*n* = 46) introduce indication‐related confounding, as treatment selection may have been influenced by patient status. Furthermore, adjustment based solely on IMDC risk may be insufficient; limited events restricted our parsimonious multivariable model from adjusting for all significant factors (e.g., age, KPS, and lymph node metastasis), leaving potential residual confounding. Without larger prospective trials, reliably determining comparative efficacy may simply be impossible, and observed survival differences should be interpreted as exploratory. Second, lacking a central pathological review, the exact percentage of sarcomatoid components could not be uniformly evaluated, potentially introducing interobserver variability. Finally, the heterogeneity of TKI agents (e.g., axitinib, lenvatinib, and cabozantinib) prevents identifying the specific contributions of individual drugs.

In conclusion, the present study is one of the few real‐world analyses that compared IO‐IO and IO‐TKI specifically for Japanese patients with sRCC. While the improvements observed in ORR and PFS were not significant, the IO‐TKI regimen showed a significant OS benefit over IO‐IO. However, given the limitations inherent to the retrospective design and small sample size, reliably determining the comparative efficacy of these regimens remains challenging. Nevertheless, the present findings suggest that IO‐TKI may represent a promising therapeutic option with a potential survival advantage for managing this aggressive variant.

## Author Contributions

**Keita Tamura:** conceptualization, formal analysis, visualization, writing – original draft, writing – review and editing. **Yuto Matsushita:** data curation, methodology. **Hiromitsu Watanabe:** data curation, methodology. **Takafumi Yanagisawa:** data curation, investigation. **Keiichiro Mori:** investigation, data curation. **Hirofumi Morinaka:** data curation, investigation. **Kensuke Bekku:** data curation, investigation. **Shingo Toyoda:** investigation, data curation. **Takuhisa Nukaya:** data curation, investigation. **Ryoichi Maenosono:** investigation, data curation. **Kazumasa Komura:** project administration, supervision. **Motoo Araki:** project administration, supervision. **Kazutoshi Fujita:** project administration, supervision. **Kiyoshi Takahara:** project administration, supervision. **Haruhito Azuma:** supervision. **Teruo Inamoto:** conceptualization, supervision, writing – review and editing.

## Ethics Statement

This study was approved by the ethics committee of Hamamatsu University School of Medicine (approval no. 25–034).

## Consent

The authors have nothing to report.

## Conflicts of Interest

The authors declare no conflicts of interest.

## Supporting information


**Figure S1:** Kaplan–Meier curves for overall survival stratified by IMDC risk category and treatment regimen. Survival outcomes were analyzed according to the IMDC risk category (Intermediate vs. Poor) and the treatment combination (IO‐IO vs. IO‐TKI). **Black line:** Intermediate risk treated with IO‐IO (*n* = 8); **Red line:** Poor risk treated with IO‐IO (*n* = 10); **Green line:** Intermediate risk treated with IO‐TKI (*n* = 19); **Blue line:** Poor risk treated with IO‐TKI (*n* = 6).

## Data Availability

The data that support the findings of this study are available from the corresponding author upon reasonable request.
