# Supplementary figures and images for: Comparative Efficacy of First‐Line Immune Checkpoint Inhibitor‐Based Combination Therapies in Patients With Sarcomatoid Renal Cell Carcinoma: A Japanese Multicenter Cohort Study
Source: Int J Urol. 2026 May 9;33:e70494. doi: 10.1111/iju.70494 (PMC13157525; doi:10.1111/iju.70494)

Supplementary Figure 1

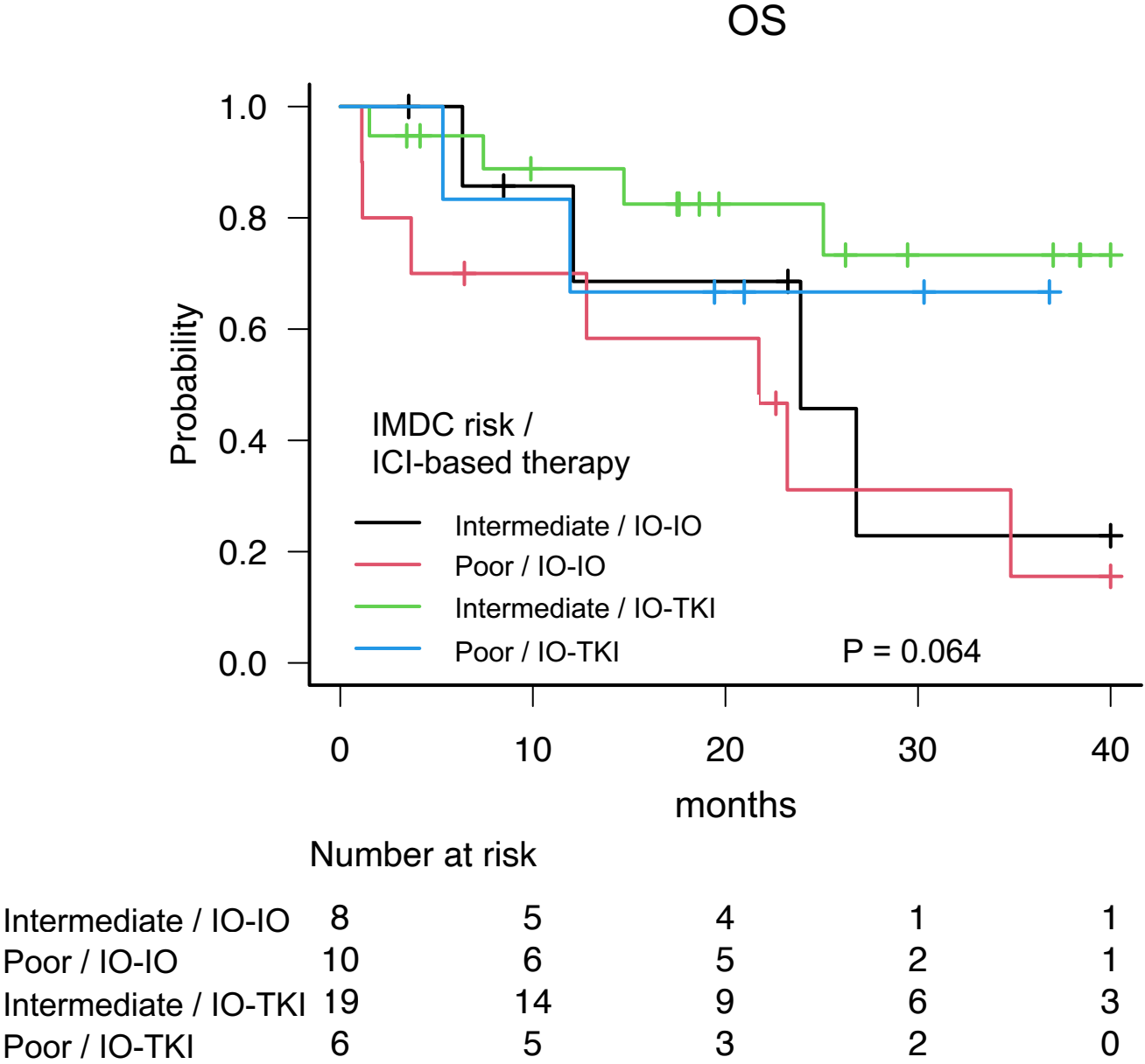

Supplement: Supplementary file 1 — Figure S1: Kaplan–Meier curves for overall survival stratified by IMDC risk category and treatment regimen. Survival outcomes were analyzed according to the IMDC risk category (Intermediate vs. Poor) and the treatment combination (IO‐IO vs. IO‐TKI). Black line: Intermediate risk treated with IO‐IO (n = 8); Red line: Poor risk treated with IO‐IO (n = 10); Green line: Intermediate risk treated with IO‐TKI (n = 19); Blue line: Poor risk treated with IO‐TKI (n = 6). [file IJU-33-0-s001.pdf]
